# Effects of probiotics in patients with diabetes mellitus type 2: study protocol for a randomized, double-blind, placebo-controlled trial

**DOI:** 10.1186/1745-6215-14-195

**Published:** 2013-07-04

**Authors:** Majed S Alokail, Shaun Sabico, Yousef Al-Saleh, Nasser M Al-Daghri, Khalid M Alkharfy, Paul M Vanhoutte, Philip G McTernan

**Affiliations:** 1 Biomarkers Research Program, King Saud University, Riyadh, 11451, Saudi Arabia; 2 Center of Excellence in Biotechnology Research, King Saud University, Riyadh, 11451, Saudi Arabia; 3 College of Medicine, King Saud University for Health Sciences, Riyadh, 11451, Saudi Arabia; 4 College of Pharmacy, King Saud University, Riyadh, 11451, Saudi Arabia; 5 Department of Pharmacology and Pharmacy, Li Ka Shing Faculty of Medicine, 21 Sassoon Road, Hong Kong, China; 6 Division of Metabolic and Vascular Health, Warwick Medical School, Clinical Sciences Research Laboratories, University Hospital Coventry and Warwickshire, Walsgrave, Coventry, CV2 2DX, United Kingdom; 7 Biochemistry Department, College of Science, King Saud University, PO Box, 2455, Riyadh, 11451, Kingdom of Saudi Arabia

**Keywords:** Type 2 diabetes mellitus, Endotoxin, Microbiota, Probiotics

## Abstract

**Background:**

Low grade chronic inflammation is observed in patients with type 2 diabetes mellitus (T2DM). Endotoxin derived from gut bacteria may act as a potent inflammatory stimulant. Probiotics, which are believed to contain health promoting live microorganisms, may influence circulating endotoxin levels. Ingestion of live probiotic cultures may alter gut microbiota in a beneficial manner to reduce inflammation; no information is available whether or not they do so in patients with T2DM. Therefore, the aim of this study is to characterize the beneficial effects of probiotics on circulating endotoxin levels and other biomarkers related to systemic low-grade inflammation in patients with T2DM.

**Methods:**

One hundred and twenty consenting adult Saudi T2DM patients (naïve or newly diagnosed and without co-morbidities) will be enrolled in this clinical trial and randomized to receive daily placebo or probiotics (Ecologic®Barrier) for 26 weeks in a double-blind manner. Inflammatory and metabolic markers will be measured and fecal samples analyzed. Measurements/samples will be obtained at baseline and after 4, 8, 12/13 and 26 weeks of treatment.

**Discussion:**

It is expected that the probiotic product will induce beneficial changes in gut microbiota, reduce the systemic inflammatory state through altering systemic endotoxin levels and, as such, reduce the systemic inflammatory response observed in T2DM subjects.

**Trial registration:**

ClinicalTrials.gov Identifier: NCT01765517

## Background

The human gastrointestinal tract contains bacteria, archaea and eukarya. In particular, bacteria living in the gut achieve the highest cell density recorded for any ecosystem [1]. However, gut microbiota does not exist independently and interacts at several levels in the functions of the body [1]. Specifically, gut microbiota can influence intestinal peristalsis [2], but also affects the expression of various host genes that regulate nutrient uptake, metabolism, angiogenesis, mucosal barrier function, the development of the enteric nervous system and maturation of mucosal immunity [3]. Interestingly it appears that fragments of Gram negative bacteria derived from the gut (endotoxin) can cross the intestinal mucosa to enter the circulation, and may represent an important mediator of low-grade systemic inflammation influenced by the host's own gut microbiota and metabolic state. Previous studies have shown that low levels of endotoxin can enter the blood circulatory system, which may stimulate an innate immune response from adipose tissue, liver and skeletal muscle, leading to production of pro-inflammatory cytokines [4-9]. Endotoxin has been shown to act as a mediator of inflammation in liver disease [10,11], whilst cross-sectional and longitudinal studies have indicated that a reduction in circulating endotoxin leads to a reduction in inflammatory cytokines and inflammatory response [12-15]. Furthermore, abnormal alterations in diet have a profound influence on human endotoxin circulation, but more so for patients with type 2 diabetes mellitus (T2DM) [16]. It makes sense that manipulation of diet to normalize the gut microflora and metabolic functions has been the mainstay management for most chronic diseases. In fact, a well-controlled diet is a major non-pharmacologic option for the prevention of T2DM [17,18]. As such, influencing the gut microbiota with the addition of probiotics may affect the systemic effects of endotoxin and represent a new and enhanced mechanism to treat low-grade chronic inflammatory conditions, such as seen in T2DM. The present study proposes to test the hypothesis that probiotics will alter the gut microbiota sufficiently to reduce systemic endotoxins and the resulting inflammation in subjects with T2DM, thus offering an additional intervention to treat these patients. To achieve this, 120 consenting adult Saudi T2DM patients without co-morbidities will be enrolled in this clinical trial and randomized to receive twice-daily placebo or probiotics for 26 weeks in a double-blind manner. Glycemic and inflammatory markers will be measured at baseline and at weeks 8, 12 and 26. The study will specifically explore the effects of probiotics on endotoxin levels in patients with DMT2 and relate them to the circulating levels of inflammatory cytokines.

## Methods

### Design

In this 26-week, single-center, double-blind, randomized, placebo-controlled study, 60 patients with T2DM will be treated with probiotics and 60 will be treated with placebo. Interventions will be performed at weeks 0, 4, 8, 12 and 26 in all subjects. Patients allocated to the probiotics group will receive two sachets with 2 g freeze-dried powder of the probiotic mixture Ecologic®Barrier daily (Winclove probiotics, the Netherlands). Ecologic®Barrier (2.5 × 10^9^ cfu/gram) contains the following bacterial strains: *Bifidobacterium bifidum* W23, *Bifidobacterium lactis* W52, *Lactobacillus acidophilus* W37, *Lactobacillus brevis* W63, *Lactobacillus casei* W56, *Lactobacillus salivarius* W24, *Lactococcus lactis* W19 and *Lactococcus lactis* W58. Participants in the placebo group will receive the placebo consisting of the carrier of the probiotic product, that is maize starch and maltodextrins. The placebo is indistinguishable in color, smell and taste from the probiotic sachets, but contains no bacteria.

### Recruitment of patients

Recruitment of patients to this study will be made possible by collaboration with primary care centers throughout Riyadh and the Biomarkers Research Program, College of Science, King Saud University.

### Ethical approval

The study protocol has been approved by the ethics committee of the College of Science, King Saud University, Riyadh, Saudi Arabia.

### Inclusion criteria

•Stable and well controlled T2DM (glycosylated hemoglobin (HbA1c) < 7.5% and no change in oral anti-diabetic medications during the last 6 months)

•Age 20 to 75 years

•Provision of written informed consent

### Exclusion criteria

•Chronic gastrointestinal disease (except irritable bowel syndrome)

•Systemic antibiotics within 6 weeks before inclusion

•Use of probiotics within 3 months before inclusion

•Regular intake of insulin or insulin analogs, antibiotics or probiotics, antacids, H2-receptor blockers, proton pump inhibitors, loperamide, cholestryramine, ω3-unsaturated fatty acid supplements, fibrates, corticosteroids or sex steroids

•Daily alcohol consumption > 30 g

•Significant immunodeficiency

•Known cardiac valvular disease

•Breast-feeding or pregnancy

•Non-Arab ethnicity

•Participation in another clinical trial within the last 6 months

•Legal incapability or mental incapacity to give consent.

### Allocation to treatment

After confirmation of eligibility and obtaining written informed consent, the patients will be given a unique subject number by the research nurse. The randomization (stratified for gender) will be performed by the probiotic company and the patients and clinicians at the primary care center will be blinded to the treatment received. The eligible patients will be allocated (1:1) to treatment for 26 weeks with either the probiotics supplement or placebo. Subjects will be instructed to:

•Ingest one sachet before breakfast and one sachet before going to bed, by mixing the powder in lukewarm water and drinking it.

•The sachets will be stored at room temperature.

### Data handling and record keeping

Case report forms (CRF) will be used to record data for all participants, and will be completed by the research nurse, who will also enter the data into an electronic database.

### Study schedule and location

After inclusion, all further treatments will be managed at the primary care center where the subject was recruited (see Table 1 for scheduled visits). A research nurse and a research dietician will be responsible for all contacts with patients. Doctors associated with the research team will be available if problems arise.

**Table 1:** Schedule of visits

	**Pre-screening**	**Screening**	**Inclusion**	**Week**				
				**0**	**4**	**8**	**12**	**26**
Patient attends the health clinic		X		X		X	X	X
Telephone review					X			
Identification of eligible subjects	X							
Check eligibility		X						
Obtain informed consent			X					
Blood sampling				X		X		X
Stool sampling				X		X		X
Provision of test product				X		X	X	
Return of previously provided test product						X	X	X
Assessment of compliance and adverse events					X	X	X	X

### Acquisition of clinical data and assessment of compliance

•Medical history, including the presence of chronic diseases, regular medication, smoking and alcohol habits, will be recorded before inclusion.

•Nutritional habits will be assessed using a standardized 14-day recall questionnaire, which will be discussed with a dietician.

•Changes in medication during the study period will be recorded

•Anthropometric measurements will be made using standardized methods. Height will be measured at the start of treatment. Weight (in light clothing without shoes or items in pockets), waist circumference (measured as a horizontal plane at the level of the umbilicus) and hip circumference (measured as a horizontal plane at the level of the trochanter major) will be measured at the start and end of the treatment.

•Liver ultrasonography will be performed either at screening or at the start of treatment to determine the presence or absence of a bright liver indicative of steatosis. Patients with fatty liver and liver transaminases >1.5 × the upper limit of normal will be offered referral to a hepatologist.

•Compliance will be monitored at weeks 4, 8, 12 and 26. Patients will bring their previously received sachets to the primary care center to calculate the mean number of sachets used per day.

### Acquisition of routine biochemical data and biological samples

•Blood samples obtained will be analyzed at the Biomarkers Research Program to measure glucose, HbA1c, insulin, C-peptide, total cholesterol, high-density lipoprotein cholesterol, low-density lipoprotein cholesterol, triglycerides, alanine aminotransferase and aspartate aminotransferase levels.

•Fasting blood samples will be collected at weeks 0, 8 and 26. Peripheral venous blood will be drawn into pyrogen-free tubes without any anticoagulant. The tubes will be immediately placed in ice, allowed to coagulate, and centrifuged (2500 ×*g* for 10 minutes at 4°C) within 2 hours of collection. Serum samples will be stored at −80°C until use. At least two 2-mL serum samples will be collected at each time-point.

•Stool samples will be obtained at the same time points as blood samples and stored in a plastic sealable bag to be handed over to the clinic. Once at the clinic, the samples will be divided, flash frozen, and stored at −80°C. Changes in gut microbiota will be profiled using signature-tagged sequencing (pyrosequencing) of amplified 16*S* rRNA genes followed by automated bioinformatics analysis. Targeted RT-PCR will also be used to examine bacterial species such as *Enterobacteria* and *Bifdobacteria* relative to the universal bacterial primer set. Pyrosequencing and RT-PCR will be performed in a sub-cohort (n = 15) of patients considering the final systemic findings. Samples will be retained for subsequent studies.

### Subject withdrawal

Subjects may withdraw from the trial at any time at their own request, or they may be withdrawn at any time at the discretion of the investigator for safety, behavioral or administrative reasons. The subjects will also be withdrawn from the study in case of:

•Treatment with systemic antibiotics for more than 1 week during weeks 0 to 22

•Any use of systemic antibiotics during weeks 22 to 26

### Patient safety

#### Adverse events

Adverse events are undesirable signs or symptoms that occur during the study and may or may not be causally related to the treatment. All adverse events considered possibly, probably or definitely related to the tested product will be recorded on CRFs.

#### Serious adverse events

Serious adverse events are defined as events that are fatal, life-threatening, disabling, incapacitating or result in hospitalization or prolonged hospital stay, or result in malformation. All will be recorded in the CRF, whether they are related to the test product or not. According to previous studies [9], probiotics are safe, and any serious adverse event that might be possibly, probably or definitely related to the test product will be regarded as unexpected. All unexpected serious adverse events will be reported immediately to the Medicines and Healthcare products Regulatory Agency. Any serious adverse event that might be related to the tested product will immediately lead to discontinuation of the test product.

### Ethical and regulatory aspects

The study will be performed in accordance with the protocol and the ethical principles that have their origin in the Declaration of Helsinki as well as with the International Council for Harmonization Guidance on Good Clinical Practice. These documents state that the informed consent of subjects is an essential precondition for participation in the clinical study. The study will commence only after obtaining approval from the local ethical review board of the College of Medicine Research Center, King Saud University.

### Statistical aspects

#### Sample size

Preliminary data indicate that serum endotoxin levels in T2DM patients are two to three times higher than in control subjects. We hypothesize that treatment with probiotics will reduce mean endotoxin levels by 25 to 30%, while no change will be seen in the placebo group. To obtain 80% power to demonstrate a statistically significant difference (two-sided *P*-value = 0.05) between the two treatments, 100 patients must be treated (50 patients per group). As we expect a dropout rate of 15%, we will include 120 patients in the study.

The sample size was calculated based on the estimated mean change during treatment (Δ-values) and corresponding standard deviation (SD) of the change. Since we have no repeated measurements of endotoxin in the same individuals, we need to estimate the SD for the change. Assuming that the correlation between 1 and 2 yields a measurement of 0.70, the SD for the Δ-value is 78% of the SD of separate measurements (Table 2). Table 3 shows the estimated sample size according to various assumptions of treatment effect and correlation between one and two measurements.

**Table 2:** Standard deviation and correlation between measures

**SD for each measurement**	**Correlation between measures**	**SD of the Δ-value**
1	0.60	0.89
1	0.65	0.84
1	0.70	0.78
1	0.75	0.71
1	0.80	0.63

Δ-value, mean change; SD, standard deviation.

**Table 3:** Sample size determination for endotoxin

**Endotoxin baseline (U/L)**	**Difference in Δ-value (probiotics –placebo)**	**SD for change (common for both treatments)**	**Correlation for 1 and 2 measurements**	**Sample size (total) 2-tailed α: 0.05 Power: 80%**	**Sample size (total) 2-tailed α: 0.05 Power: 90%**
10 (± 6)	−2	5.34	0.60	224	298
	−2	5.04	0.65	200	266
	−2	4.68	0.70	176	236
	−2	4.26	0.75	148	198
	−2	3.78	0.80	116	154
	−2.5	5.34	0.60	144	192
	−2.5	5.04	0.65	128	172
	−2.5	4.68	0.70	114	152
	**−2.5**	**4.26**	**0.75**	**96**	**128**
	−2.5	3.78	0.80	76	100
	**−3**	**5.34**	**0.60**	**100**	**134**
	−3	5.04	0.65	90	120
	−3	4.68	0.70	80	106
	−3	4.26	0.75	68	90
	−3	3.78	0.80	54	70
	−4	5.34	0.60	58	76
	−4	5.04	0.65	52	68
	−4	4.68	0.70	46	62
	−4	4.26	0.75	40	52
	−4	3.78	0.80	32	40

Δ-value, mean change; SD, standard deviation.

### Plan for data analysis

As this trial is designed to assess the physiological effects rather than support clinical indications for treatment with probiotic**s** in T2DM, we plan to perform per protocol analyses. Only subjects treated with at least 80% of the planned doses for at least 80% of the time will be considered as treated per protocol.

Raw data will be entered into statistical software (SPSS version 16.5, Chicago, IL, USA). Preparation of the dataset will be done prior to analysis. Descriptive statistics will be performed, and frequencies will be presented as a percentage. Continuous variables will be presented as mean ± SD. Variables exhibiting non-Gaussian variables will be transformed prior to analysis. Repeated measures analysis of co-variance will be used to compare groups, which will represent a combination of analysis of variance and linear regression. Confounding and fixed variables such as age and gender, as well as body mass index, will be taken if proper matching has not been achieved prior to repeated measures. *Post hoc* Banjamini-Hohberg corrections will be done for multiple comparisons. For comparison of two groups, an independent *t*-test will be applied for continuous variables and Mann–Whitney for non-continuous variables. Analysis of adverse events (if any) will be performed using Chi-Square test and Fisher’s exact test following assumptions of randomness, independence and size. Significance will be set at *P* < 0.05.

### Limitations

The investigators are aware that systemic inflammatory markers may not change during the clinical trial; however, this would not preclude the study as we understand that probiotics do affect gut microbiota and that this may have beneficial effects as noted in previous rodent and human studies. Furthermore, probiotics have been previously shown to affect lipopolysaccharide levels and this may still have long-term beneficial effects which may have a trend effect on inflammatory or insulin resistance markers and thus may indicate that a longer term treatment is required. In either case, to examine the effects of a probiotic in a clearly designed clinical trial will provide new understanding and warrants investigation.

## Discussion

Many different probiotic strains have been identified and the effects of these bacteria, either given in monoculture or as a cocktail of various strains, have been subject to increasing scientific evaluation in recent years. Probiotic bacteria (with the best documented strains being species of *Lactobacillus* or *Bifidobacterium*) inhibit the growth of pathogenic bacteria by acidifying the gut lumen, competing for nutrients, and producing antimicrobial substances [19,20]. Furthermore, they adhere to the gastrointestinal mucosa and are thought to prevent bacterial translocation from the gut [21]. The strongest evidence for the use of probiotics has been in the management of diarrhea [22]. Data extrapolated from a large number of studies, including systemic reviews [23-26], meta-analyses [27-30], open-label studies [31,32] and multicenter trials testing the efficacy of probiotics in preventing diarrhea, concluded that, in addition to having a good safety profile, probiotics significantly reduced the duration and frequency of acute diarrhea [33]. In addition, trials have documented favorable effects of probiotics in other gastrointestinal diseases (for example, irritable bowel syndrome, pouchitis and necrotizing enterocolitis in premature infants). Studies in obesity have also shown altered gut microbial compositions in human subjects and in mice [34-43]. The guts of obese human subjects appear to have reduced numbers of *Bacteroidetes* and increased numbers of *Firmicutes* compared with lean people [36]. During a 1-year intervention trial in obese humans on a hypocaloric diet, an increased proportion of fecal *Bacteroidetes* was observed to parallel weight loss [36]. Diet-induced obesity in animal models may also lead to increased *Mollicutes* (a class of *Firmicutes*), a response which appears to be reversible with dietary manipulation aimed at limiting weight gain [37]. The finding that the microbial composition is modulated by dietary modification suggests that differences in the gut composition between obese and lean individuals are related to dietary factors independent of obesity [15,44]. However, not all studies have shown beneficial effects of probiotics, and thus caution should be taken in terms of the dosage and strains to be used, as these may have important ramifications on the effects observed. In this study, a combination of probiotic strains will be used, as there are suggestions that multispecies products are more effective than single strain products [45,46]. The strains were selected for this product based on different *in vitro* screening criteria. Among the criteria were the inhibition of cytokine-induced barrier dysfunction of the epithelial cell line Caco-2, the capacity to induce expression of interleukin-10 [47] (as this anti-inflammatory cytokine has a protective function on the epithelial barrier [48]), the ability to break down lipopolysaccharides and the inhibition of mast cell activation [49].

Taken together, the current evidence appears to support a role for the gut microbiota in the pathogenesis of diet-induced obesity and related metabolic disorders, which may be reversible with dietary and/or gut microbiota manipulation [50]. As the gut microbiota is the main source of endotoxin, treatment with probiotics may influence the circulating levels of endotoxin by altering the microbiota composition. To date, relatively few studies have examined the effects of endotoxin in metabolic diseases using probiotics. To the best of our knowledge, this hypothesis has not been tested except in a small study of patients with cirrhosis in which a 25% reduction in endotoxin was reported [1]. However, animal studies have revealed that treatment with probiotics may be beneficial in insulin-resistant states [2,37]. Probiotics also delay the onset of glucose intolerance in high-fructose-fed rats [3]. To expand on these earlier findings, the present study will explore the potential beneficial effects of probiotics on circulating endotoxin levels and other markers for systemic low-grade inflammation in patients with T2DM.

In summary, we expect that our findings will demonstrate that probiotics alter gut microbiota in T2DM and that, in theory, the alterations will be metabolically favorable with continued probiotic use. Since the efficacy of probiotics is directly linked to the type of strain, we will also be able to identify which strains used in the intervention are most beneficial for patients with T2DM. Further investigations, however, will be needed whether the efficacy of the probiotics used in this study can be extended to prevention levels. Regardless, our study will be the first to address this issue beyond classical inflammatory conditions. This will have a fundamental impact on how we should treat the inflammatory component of T2DM, particularly once the results are verified in a larger cohort of patients.

## Trial status

Estimated enrollment: 120

Study start date: October 2013

Estimated study completion date: October 2015

Estimated primary completion date: October 2015

## Abbreviations

CRF: case report form; HbA1c: glycosylated hemoglobin; RT-PCR: reverse transcription polymerase chain reaction; SD: standard deviation; T2DM: type 2 diabetes mellitus.

## Competing interests

This project is funded by the National Plan for Science and Technology (NPST) (Grant Number: 11-MED2114-02).

## Authors’ contributions

SS, MSA, YA and PGM are the principal researchers and conceptualized the study. NMA, KMA and PMV further developed the study design. SS will develop the statistical design. All authors have seen and approved the final version of the protocol.
